# Plastic Quantification and Polyethylene Overestimation in Agricultural Soil Using Large-Volume Pyrolysis and TD-GC-MS/MS

**DOI:** 10.1021/acs.est.3c10101

**Published:** 2024-07-08

**Authors:** Ryan Bartnick, Andrei Rodionov, Simon David Jakob Oster, Martin G. J. Löder, Eva Lehndorff

**Affiliations:** † Soil Ecology, University of Bayreuth, Dr.-Hans-Frisch-Str. 1-3, 95448 Bayreuth, Germany; ‡ Animal Ecology I, University of Bayreuth, Universitätsstraße 30, 95447 Bayreuth, Germany

**Keywords:** gas chromatography–tandem mass spectrometry, microplastics, polyethylene terephthalate, polystyrene, soil, thermal desorption

## Abstract

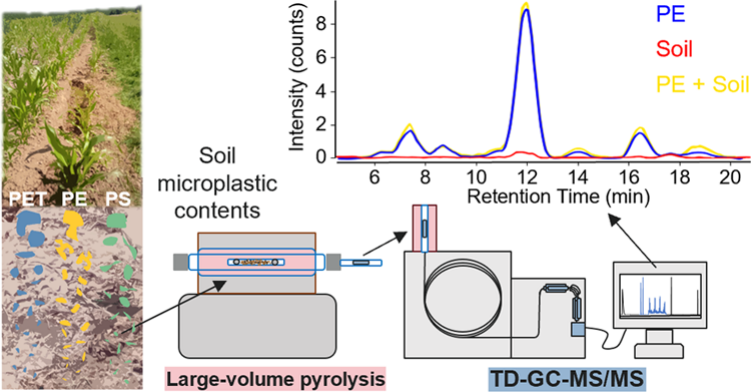

Quantification of microplastics in soil is needed to understand their impact and fate in agricultural areas. Often, low sample volume and removal of organic matter (OM) limit representative quantification. We present a method which allows simultaneous quantification of microplastics in homogenized, large environmental samples (>1 g) and tested polyethylene (PE), polyethylene terephthalate (PET), and polystyrene (PS) (200–400 μm) overestimation by fresh and diagenetically altered OM in agricultural soils using a new combination of large-volume pyrolysis adsorption with thermal desorption–gas chromatography–tandem mass spectrometry (TD-GC-MS/MS). Characteristic MS/MS profiles for PE, PET, and PS were derived from plastic pyrolysis and allowed for a new mass separation of PET. Volume-defined standard particles (125 × 125 × 20 μm^3^) were developed with the respective weight (PE: 0.48 ± 0.12, PET: 0.50 ± 0.10, PS: 0.31 ± 0.08 μg), which can be spiked into solid samples. Diagenetically altered OM contained compounds that could be incorrectly identified as PE and suggest a mathematical correction to account for OM contribution. With a standard addition method, we quantified PS, PET, and PE_corrected_ in two agricultural soils. This provides a base to simultaneously quantify a variety of microplastics in many environmental matrices and agricultural soil.

## Abstract 2

It is a continuing difficulty to analyze microplastics in soil on an environmentally relevant level. This study provides further development into microplastic detection and shows the risk of overestimating polyethylene as organic matter in soils.

## Introduction

Plastics are ubiquitous to the environment, but their representative quantification is still a challenge. In agricultural soil, microplastics are heterogeneously introduced via atmospheric inputs, fertilizers, sewage sludge, and plastic mulches^1−4^ and cover a large size range from millimeters to nanometers.^5,6^ Also, all relevant soil processes, like carbon and nutrient cycling and water retention, occur mainly in this range.^7,8^ As microscopic methods would fail to cover this,^9^ a focus on pyrolysis-based, thermal analytical quantification methods is required.^10^ However, until now, quantification is limited by soil plastic concentrations and by the need to separate from other organic matter (OM).^11^

In soils, plastic is most likely rather heterogeneously distributed, e.g., incorporation processes might lead to highly variable distribution of plastic of different sizes and types and shapes in soil pores and aggregates.^12^ A method considering the full heterogeneity of soil requires homogenization of a large sample volume, e.g., by milling and subsequent quantification of environmentally relevant plastic mixtures. Still, the concentration of plastics in agricultural soil is expected to be low in many cases,^13,14^ but current plastic quantification methods are limited to low sample amounts or require preconcentration steps.^15,16^ Often, a plastic extraction from a large sample volume is needed to cover all possible concentrations in soil, and we test whether preconcentration can be avoided when using large-scale pyrolysis of soil samples.

Pyrolysis of soil is already an established method for plastic detection. However, pyrolysis–gas chromatography–mass spectrometry (Py-GC-MS)^17^ and pyrolysis adsorption–thermal desorption–GC-MS (e.g., using a TED-GC-MS system)^18,19^ methods are still challenged by the presence of other OM. In Py-GC-MS, pyrolyzed plastics and OM would directly enter the GC-MS system. In methods based on pyrolysis adsorption–thermal desorption–GC-MS, only a portion of OM would be transferred for analysis, potentially allowing to reduce a time-consuming sample cleanup; however, a single quadrupole MS would not have the selectivity required for precise detection of every plastic type,^20^ e.g., polyethylene (PE) and polypropylene (PP). Hence, a selective cleanup for OM other than plastics has to be implemented to avoid interferences on the GC column and in the detection system. In soil matrices, a huge variety of OM are present, and methods to remove via density separation and enzymatic digestion are extensive.^21^ It is, for example, known that both PE and OM when pyrolyzed produce mono-unsaturated hydrocarbons, resulting in OM being the highest contributor to noise and interference.^22^ However, a further approach, tandem mass spectrometry (MS/MS), was also shown to improve plastic quantification by reducing interferences compared to using a single quadrupole MS in scan or selected ion monitoring (SIM) mode, as shown by Albignac et al. for PE and PP in water samples.^20^ Hence, we here explicitly tested whether an offline large-volume pyrolysis adsorption–thermal desorption–GC-MS/MS method would allow us to avoid the cleanup for quantification of soil samples from an agricultural context.

Microplastic quantification with adequate standard materials is another challenge in thermal analytical techniques. Some methods take advantage of dissolving plastics via pressurized liquid extraction;^23,24^ however, this is restricted to materials which can be dissolved and might involve strong solvents and high temperatures. Others considered isotopically labeled standard materials, which were limited to easily dissolvable polymers and might be affected by isotope exchange with the matrix during pyrolysis.^25^ The development of solid standard materials would be independent of different solubilities and, hence, be most representative of the intrinsic plastic content of an environmental sample.

In this study, we aim at developing a method that allows analyzing a representative, homogenized sample to then simultaneously quantify soil plastic contents. We hypothesized that using an offline large-volume pyrolysis adsorption–desorption method would improve representativeness, that newly developed solid standard materials facilitate quantification for various types of plastic, and that the benefit of combining large-volume pyrolysis with adsorption–thermal desorption and MS/MS will finally allow one to sufficiently quantify a variety of plastics in agricultural soil without excluding OM. To test this, we developed a new analytical setup for offline large-volume pyrolysis adsorption–thermal desorption–gas chromatography–tandem mass spectrometry (TD-GC-MS/MS) and analyzed materials with increasing complexity and potential for interference; these include lab blanks, polyethylene (PE), polyethylene terephthalate (PET), and polystyrene (PS) as well as fresh and diagenetically altered OM and agricultural soils. This method development is meant to serve as a base for further application in soil science and many other environmental research areas dealing with plastic detection in complex matrices.

## Materials and Methods

### Plastic Materials and Reference Compounds for Identification of Plastic Pyrolysis Products

For the first step, the identification of plastic pyrolysis products, we used pure plastic materials and the respective compounds resulting from pyrolysis (reference compounds).

Throughout the experimental process, contact of samples to other plastics was avoided. All materials were kept in glass containers and handled with only metal tools. The plastic polymers low-density polyethylene (LD-PE, Lupolen 1800 P-1—LyondellBasell, Rotterdam, NL), polyethylene terephthalate (PET, CleanPET WF—Veolia Umweltservice, Hamburg, Germany), and polystyrene (PS 158N/L—INEOS Styrolution, Frankfurt, Germany) were in a size range of 200–400 μm of irregular shape (see the Supporting Information, SI). Plastics were prepared by cryomilling (ZM200; Retsch, Haan, Germany) and air jet sieving (E200 LS; Hosokawa Alpine, Augsburg, Germany). Five supplemental plastics: polypropylene (PP, Moplen HP 5261—LyondellBasell, Rotterdam, NL), polyamide (PA66, Ultramid A27 E—BASF SE, Ludwigshafen, Germany), polybutylene adipate terephthalate (PBAT, M·VERA B5026—BIO-FED, Cologne, Germany), polylactic acid (PLA, Ingeo Biopolymer 7001D—NatureWorks, MN), and polymethyl methacrylate (PMMA—LLV-Shop.de, Niederkassel, Germany) were provided and analyzed.

To identify retention time (*t*_R_) and ions of interest (mass to charge, *m*/*z*) of plastic pyrolysis products, reference compounds were purchased or made to compare to plastic pyrolysis products. Reference compounds were the following—PET: vinyl benzoate, ethyl benzoate, benzoic acid, and biphenyl; PS: styrene; and PE: 1,9-decadiene and 1,13-tetradecadiene (all compounds were purchased from Sigma-Aldrich Chemie, Taufkirchen, Germany, with the exception of benzoic acid from Fluka, Buchs, Switzerland). Due to a lack of available PS dimer and trimer compounds for purchase, a solution was made by dissolving 100 mg of PS per mL of tetrahydrofuran. For quality assurance and identification, reference compounds vinyl benzoate, ethyl benzoate, and styrene were diluted in methanol to a concentration of 0.2 μg μL^–1^; benzoic acid, biphenyl, 1,9-decadiene, and 1,13-tetradecadiene were diluted to a concentration of 2 μg μL^–1^. 1 μL of reference compound was then injected through the septum of a closed thermal desorption vial directly onto a nonpolar sorbent (Sorb-Star; ENVEA, Karlsfeld, Germany) and analyzed.

### Development of Solid Standards for Quantification of Plastics

To overcome the limitations of standards for microplastic analysis, a novel production of rectangular, volume-defined standard particles (125 × 125 × 20 μm^3^) was used with an average respective weight (PE 0.48 ± 0.12, PET 0.50 ± 0.10, PS 0.31 ± 0.08 μg per particle), which can be directly introduced into solid samples for pyrolysis. For production of standard plastic particles, a protocol from Oster et al. was used.^26^ Injection-molded polymer blocks made of LD-PE, PET, and PS were cut into rectangular pieces (10 × 10 × 4 mm^3^). These were then processed using a CNC mill (CMX 600 V; DMG MORI Inc., Bielefeld, Germany) to create columns on a baseplate with the intended diameter of the particles. The columns were then embedded in gelatin, frozen at −19 °C for 10 min, and cut using a cryomicrotome (CM1950; Leica Camera Inc., Wetzlar, Germany) also operated at −19 °C. The resulting slices (20 μm thickness) were subsequently filtered with the help of a 10 μm-pore size stainless-steel filter and 60 °C filtered milli-Q water (0.2 μm-pore size cellulose acetate filters) to remove the gelatin. The standard plastics were then picked up from the filter using a tool made of a single hair attached to the tip of a pipet and transferred into vials for usage in TD-GC-MS/MS analysis.

The use of solid particles, as opposed to adding a solution of internal standard, has several advantages, e.g., steps of dissolution and reprecipitation can be avoided. Plastic particle standards are placed on glass fiber filters, analyzed under a microscope, and inserted into the sample internally for pyrolysis.

### Soil and Organic Materials

**Figure 1:**
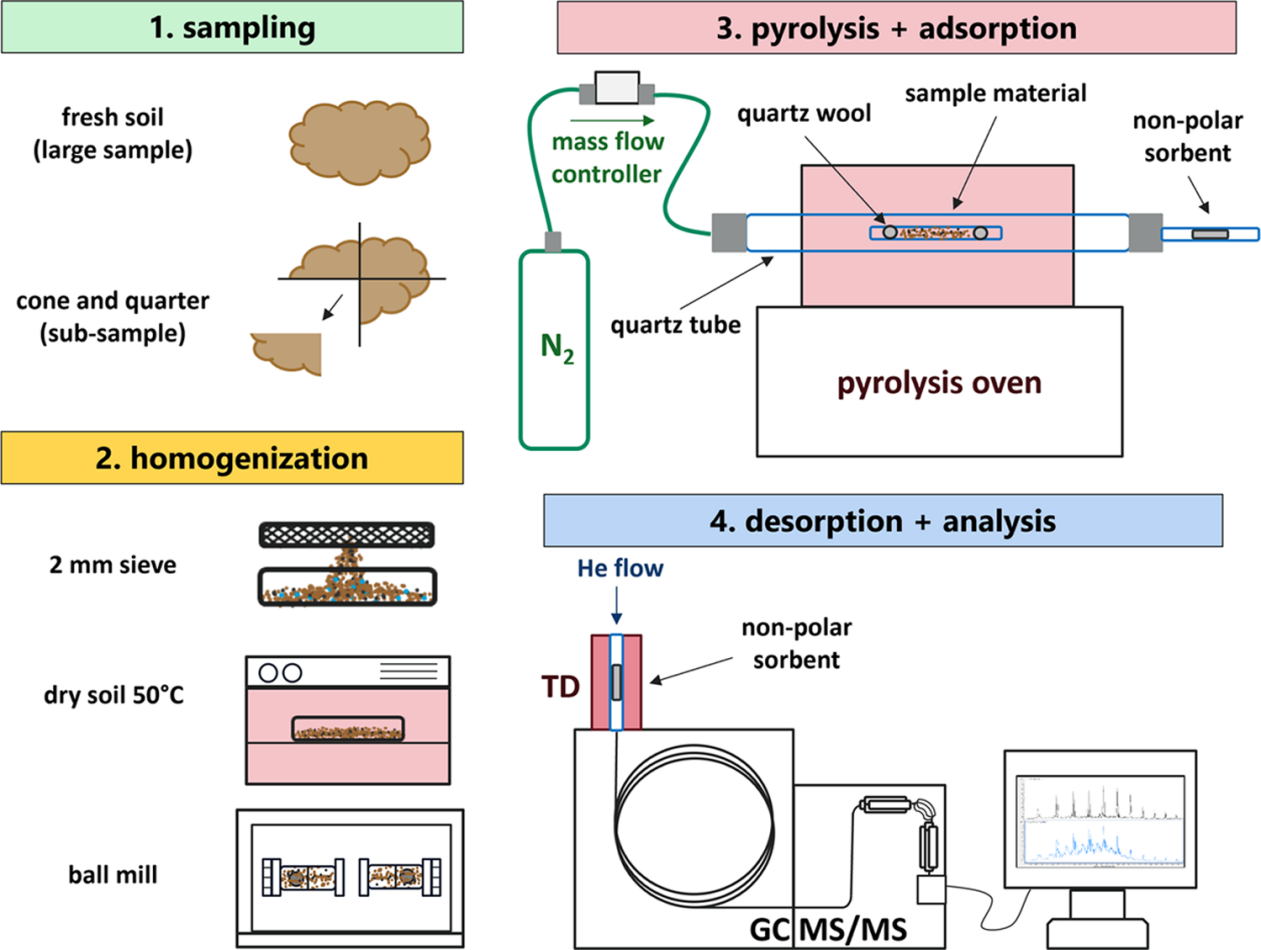
Procedural steps of sampling and homogenization, pyrolysis and adsorption, and analysis with thermal desorption–gas chromatography–tandem mass spectrometry (TD-GC-MS/MS); manual transfer of the trapped sample in the vial between adsorption and desorption steps.

Past studies have demonstrated that alkadienes are more selective for PE detection, but possible interferences from OM still persist, especially among humic acids and diagenetically altered OM.^22,23^ To check for potential signal contribution of natural organic compounds to any of the pyrolyzed plastics, we tested fresh biomass such as inner wood from a beech tree (*Fagus sylvatica*) and yeast as well as organic materials of higher maturity and kinetic stability such as leonardite (Humintech, Grevenbroich, Germany) and humic acids (Humintech, Grevenbroich, Germany; Sigma-Aldrich Chemie, Taufkirchen, Germany). As yeast, a *Komagataella pastoris* strain Pi-0702 (DSM 70382; German Collection of Microorganisms and Cell Cultures, Braunschweig, Germany) was cultured at 25 °C in minimal medium;^27^ cells were then harvested after centrifugation and freeze drying. Humic acid was obtained from raw lignite via alkaline digestion (Northern Hesse, Germany/Sigma-Aldrich Chemie, received with a standard high-density PE bottle). To avoid contamination from storage in PE bottles, leonardite, a mineraloid of oxidized lignite with a high humic acid content, was collected (North Rhine-Westphalia, Germany/Humintech, provided in glass containers with no contact to plastics). Soil samples of two agricultural fields (several kg of topsoil, 0–30 cm, of a sandy and silty soil; see Table S1, SI) were collected from Bayreuth, Germany. Subsamples were produced by mixing soil using a cone and quartering method,^28^ sieved at 2 mm, dried at 50 °C, and ground using a ball mill (MM 400; Retsch, Haan, Germany, for soil preparation, see the analytical scheme, Figure 1). Aliquots of 1 g of soil were initially analyzed for their “natural” plastic contents using a standard addition calibration method (5 replicates) and then reduced to 0.5 g aliquots if plastic contents exceeded the quantification range. To compensate for matrix effects, the standard addition method adds known concentrations of analyte to samples in increasing amounts, “spikes”, to then extrapolate the analyte signal in the sample matrix.^29^

### Large-Volume Pyrolysis

Pyrolysis was performed in a stand-alone tube furnace with a programmable temperature (Carbolite Gero TF1-1100; Verder Scientific, Haan, Germany). All equipment was precleaned, quartz glass and sand (900 °C, 3 h), glass wool and fiber filters (550 °C, 3 h), sorption vials, and septa (300 °C, 2 h). Samples for pyrolysis were inserted into a quartz sample tube (4 mm inner diameter (i.d.) × 6 mm outer diameter (o.d.) × 100 mm) and fitted with two balls of glass wool on each end to retain the sample. The sample tube was then inserted inside a larger quartz pyrolysis tube (7 mm i.d. × 9 mm o.d. × 400 mm; heating volume: 3.85 cm^3^), which was held inside the pyrolysis oven. Another glass tube of a specific length was used to push the sample tube directly into the middle of the pyrolysis oven to ensure equal heating throughout the sample. The large quartz tube was then connected with metal Swagelok connectors (Swagelok Company, OH), with one end to a N_2_ carrier gas (99.999%) flow line and the other end to a sorption tube fitted with a Sorb-Star (a polydimethylsiloxane bar with a large surface to trap nonpolar, semivolatile pyrolysis products; see the analytical scheme, Figure 1). Early tests optimizing the pyrolysis flow and heating rates showed reproducibility of the plastic peaks PS, PET, and PE (Figure S1, SI), before accounting for lab blank signal contributions. The tube furnace heating program was from 25 to 600 °C at a rate of 15 °C min^–1^, then held at 600 °C for 30 min, and flushed with a constant N_2_ flow of 8 mL min^–1^ from the sample toward the sorbent during pyrolysis (all parameters tested and optimized as adapted from previous TED-GC-MS applications).^18,19,30^ As Dümichen et al. (2014) state, there is no optimum flow rate to cover all polymer applications and a compromise has to be chosen; early testing of our method showed that a flow rate of 8 mL min^–1^ was optimal in our system for peak sensitivity and loading time on the sorbent. The heating ramp was increased from 10 to 15 °C min^–1^ to save time efficiency without affecting peak intensity. The sorption tube consisted of an open glass tube aligned with a Sorb-Star lying flat inside the middle of the tube in the direction of the gas flow; nonadsorbed materials were vented. The sorption tube was disconnected after pyrolysis and closed with polytetrafluoroethylene (PTFE) septa and an aluminum cap on the top and bottom. The pyrolysis and sorption systems were cleaned by replacing all glass tubes.

### Thermal Desorption–Gas Chromatography–Tandem Mass Spectrometry

Pyrolysis products of plastics were detected and quantified by thermal desorption–gas chromatography–tandem mass spectrometry (TD-GC-MS/MS) using a PAL autosampler with a Chromtech thermal desorption unit (PAL3 RSI TDAS 2020; Chromtech, Bad Camberg, Germany) coupled to a gas chromatograph with a tandem quadrupole mass spectrometer (Agilent 7890B plus 5977B modified to a Chromtech Evolution 3; Chromtech, Bad Camberg, Germany). The GC (fused) silica capillary column was a Macherey-Nagel OPTIMA 5 MS (30 m × 0.25 mm i.d. × 0.25 μm film thickness, split injection: 1:100, inlet temperature: 300 °C, He gas flow: 1 mL min^–1^).

Before injection into the GC, the sorption tube was flushed with N_2_ to remove any gases from the headspace and then transported inside a preheated TD unit for desorption at 300 °C for 5 min. During this, compounds adsorbed to the sorbent are desorbed into the gas phase and, at the end, injected to the GC column via a helium gas flow (followed by 5 min of flushing of the injection system in heated mode). The GC oven temperature program was set to standby at 40 °C for 1 min, ramp to 285 °C at 7 °C min^–1^, and postrun at 320 °C for 5 min (GC parameters tested and optimized for time efficiency and peak separation, as adapted from previous GC-MS applications).^18,19,23^

The detection system was used either in single quad full scan mode (45–450 *m*/*z*) or selected ion monitoring mode (SIM, target ions see Table 1) or in triple quad (tandem MS) selected reaction monitoring (SRM) mode (electron ionization at 70 eV, ion source temperature: 230 °C, quadrupole 1 at 150 °C, mass resolution: ±1.0 *m*/*z* in quadrupole 1 and 3, collision energy: −10 V, targeted mass fragments, see Table 1).

**Table 1:** Pyrolysis Product Compounds of Polyethylene Terephthalate, Polyethylene, and Polystyrene Plastic Polymers^a^

						SRM (*m*/*z*)	
polymer label	compound (pyrolysis product)^b^	*t*_R_ (min)	molecular formula	molar mass	SIM (*m*/*z*) Q1	Q1	Q3
PS1	styrene	5.4	C_8_H_8_	104	104	104	78
PET1	vinyl benzoate	12.2	C_9_H_8_O_2_	148	105	148	105
PET2	ethyl benzoate^c^	12.8	C_9_H_10_O_2_	150	105	150	122
PET3	benzoic acid	13.0	C_7_H_6_O_2_	122	105		
PE1	1,12-tridecadiene	15.4	C_13_H_24_	180	81	81	79
PET4	biphenyl	17.3	C_12_H_10_	154	154	154	152
PE2	1,13-tetradecadiene^c^	17.4	C_14_H_26_	194	81	81	79
PE3	1,14-pentadecadiene^c^	19.2	C_15_H_28_	208	81	81	79
PE4	1,15-hexadecadiene	20.9	C_16_H_30_	222	81	81	79
PS2	2,4-diphenyl-1-butene^c^	23.2	C_16_H_16_	208	91	208	104
PS3	2,4,6-triphenyl-1-hexene	32.9	C_24_H_24_	312	91	312	207

a Ordered by the Corresponding Retention Time, *t*_R_.

b Characterized by selected reaction monitoring (SRM) ions of interest (*m*/*z*) at quadrupole 1 (Q1) and quadrupole 3 (Q3) of the tandem mass spectrometer.

c Used for quantification.

### Quality Control and Quantification

To account for the lab background signals, a blank was estimated from glass wool, filters, and the N_2_ carrier flow during pyrolysis (*n* = 18 replicates). We defined the blank offset as the average target response of the blank samples. The blank offset was determined as the *y*-axis origin for calibration (see Table S2, SI). Calibrations were made using plastic mixes weighed on a cut piece of glass fiber filter, inserted into a quartz sample tube, and pyrolyzed to test for the linearity of the signal and limit of quantification (MassHunter Quantitative Analysis software; Agilent, CA). Microplastic contamination is present in nearly all laboratory analyses, and thus, the limit of quantification (LOQ) is defined by the lab background signals and not the analytical instrument. A calibration was made with our novel defined plastic particles and weights of approximately 0.5–250 μg (*n* = 20, each plastic mixed) to determine the lowest amount standard that could be reliably detected above the lab background signal (see Table S2, Figure S2, SI). For testing detectable concentrations ranging over multiple levels of magnitude as can be expected for agricultural soil, a calibration of approximately 150–850 μg (*n* = 10, each plastic mixed) was plotted with the lower concentration range as a double log plot to correlate a linear function over several orders of magnitude (see the Results and Discussion section and Figure S3, SI).

Organic substances (triplicates, 20 mg, representative of a typical amount of 2% OM in topsoil) were tested for potential contribution to characteristic signals of plastic pyrolysis products of PS, PE, and PET. For quantification of organic contributions, we used the calibration of pure plastic standard particles (0.5–250 μg). Only for characteristic PE pyrolysis products, i.e., tetradecadiene and pentadecadiene, interferences from OM were found using the method described below. Calculation of potential OM contribution to such alkadienes was estimated from the mass detector response of humic acid, leonardite, wood, and yeast and compared it to the response of the pure PE pyrolysis product (alkadiene_PE_) (*PE*_overestimation factor_, eq 1).
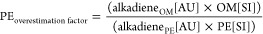
1For this study, we averaged the PE overestimation factor using tetradecadiene from humic acid and leonardite (see Table S3, SI). Yeast and wood were not considered as they were tested to be under the previously determined LOQ. The use of pentadecadiene is discussed below. We calculated a PE correction for soil samples (PE_corrected_, eq 2) using the PE overestimation factor, sample amount, and soil OM contents, soil organic carbon (OC), from elemental analysis multiplied by a factor 2 to account for other elements in OM.
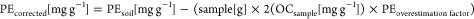
2For PE overestimation from OM, we later discuss whether this can be specified for only the recalcitrant OM portion in soil. In soils, solid standards of PE, PS, and PET were used for standard addition to quantify the respective plastic types and account for matrix effects (soil initially spiked with the expected plastic content of 0.01% and then adjusted to spike at 1× and 2× the estimated plastic content of each polymer; see Figure S4, SI).

## Results and Discussion

### Method Development—MS/MS Parameters

**Figure 2:**
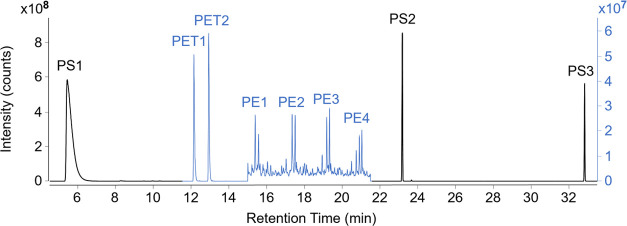
Chromatogram of polystyrene (PS1–3, black scale), polyethylene terephthalate (PET1–2, blue scale), and polyethylene (PE1–4, blue scale) pyrolysis products using selected reaction monitoring (SRM) mode of MS/MS (100 μg each plastic PE, PET, PS; peak names refer to pyrolysis products of each plastic, see Table 1).

Characteristic pyrolysis products from PE, PET, and PS were identified by the respective reference polymer compounds to verify retention times and ions of interest. We selected pyrolysis products which had the lowest risk to coeluate with pyrolysis products from other plastics. First, we could confirm characteristic ions suggested for full scan mode analysis of the mass spectra.^19^ Second, a selected ion monitoring (SIM) method was established to set quadrupole 1 (see SIM *m*/*z* Q1, Table 1) to the ion with the best selectivity to the specific pyrolysis product, also informed by the literature.^18,19,23,31−33^ Third, a product ion scan (PIS) method was used to have another reaction step in a second quadrupole (Q2) with N_2_ collision gas and then a scan of the product ions generated at a third quadrupole (Q3). Finally, the selected reaction monitoring (SRM) method was completed for each pyrolysis product (see SRM *m*/*z* Q1 and Q3, Table 1, Figure 2; compounds identified by the mass spectrum from the NIST online library^34^).

**Figure 3:**
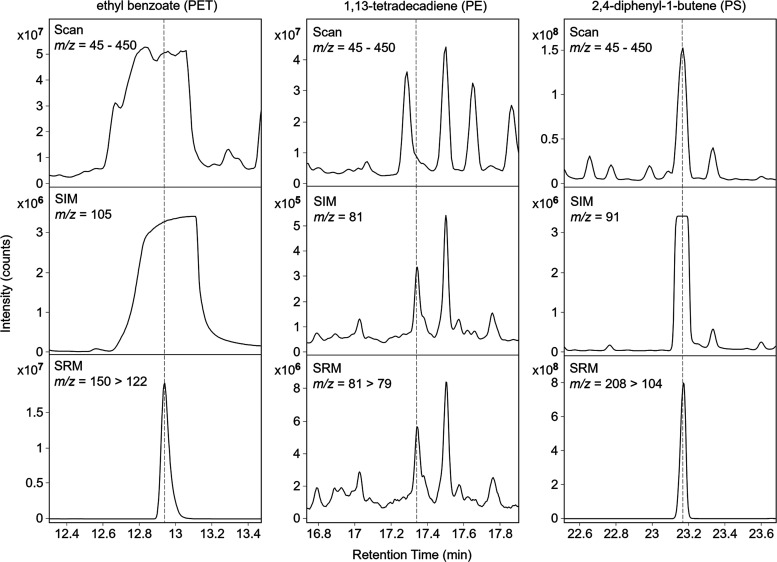
Chromatograms of pyrolyzed products of plastics PE, PET, and PS, 50 μg each. Increasing precision and intensity of signal counts is observed transitioning from MS modes of scan to selected ion monitoring (SIM) and then selected reaction monitoring (SRM) mode of MS/MS.

The pyrolysis products found here (Table 1) agreed well with the previous literature.^18,19^ However, for PET, we were able to improve the peak separation of ethyl benzoate from benzoic acid, which elutes at the same time (Figure 3). During SIM mode, ion 105 is commonly used to detect many PET (PET1–3) compounds, as this ion has the highest response. However, we were now able to separate ethyl and vinyl benzoate by mass from the lower weight benzoic acid, which fronts on the column over ethyl benzoate, by setting Q1 to the entire molar mass of these compounds (see SRM *m*/*z* Q1, Table 1). Then, Q3 was set to the highest product ion produced from the Q1 mass separation, creating excellent separation of ethyl benzoate from benzoic acid, which was chosen as the calibration compound for PET over vinyl benzoate due to higher response. It should be noted that PET pyrolysis additionally produces biphenyl (PET4), a compound which could potentially be used to quantify PET; however, its retention time overlapped the signal of PE2, so it was excluded from analysis.

For PE, pyrolysis yields several alkenes and alkadienes, which elute across much of the chromatogram runtime, resulting in many peaks with relatively low intensity. Dümichen et al. used ion 55 to detect alkenes and alkadienes from PE pyrolysis products, but they also found significant contributions to alkenes from OM.^18^ Albignac et al. used ion 95; however, we focused on ion 81, which had more intensity for alkadienes (see Q1, Table 1).^20^ Of the four alkadienes (PE1–4) with the highest signal intensities from PE pyrolysis, we selected 1,13-tetradecadiene (PE2) as the calibration compound because it had the highest peak response and had an available reference compound. While separation of PE1–4 from other compounds is comparable between SIM and SRM modes (see Figure 3), the benefit of utilizing a second mass detector is a significantly higher signal intensity for environmental samples (see the degree of intensity counts, Figure 2). The benefit of MS/MS application is a higher detector sensitivity due to reduction of untargeted ions.

For PS, we first observed carryover effects that could be minimized by increasing conditioning temperature in the TD unit (from 200 to 300 °C) and running blanks between samples for cleaning needles. PS pyrolysis products of styrene monomer, dimer, and trimer can all produce carryover effects in the TD-GC part of the system if there is inadequate postrun heating. Especially, the heavy styrene trimer can potentially stick in the system between the TD unit and the GC inlet wherever there are unheated areas of the needles. For PS quantification, the monomer styrene (PS1) could not be recommended because it is a pyrolysis byproduct of lignin found in soil.^16^ Therefore, the dimer, 2,4-diphenyl-1-butene (PS2), was selected as the calibration compound with the highest peak response for PS, which is well separated from OM and agrees to the literature.^18,19,35^ These authors used ion 91 for quantification; however, the signal is too intense in tandem MS when targeting other plastic simultaneously. Therefore, we first fragmented the dimer, excluded *m*/*z* 91, and sent the remaining parent molecules (*m*/*z* 208) to further fragmentation (*m*/*z* 104) (Figure 3). We thus reduced the signal of styrene and improved its simultaneous detection with other types of plastics.

### Method Development—Quantification of MP

Plastic concentrations in the environment may cover a large range, from extremely low to spots with high accumulation. Since plastics occur ubiquitously, we recommend to start with accounting for the lab background signal.^23^ For our calibration curves, the lab background signal defined the *y*-axis origin of slope and was removed from contributing to the calibration (“blank offset”, Table S2, SI). To further check a range of concentrations, a series of PE, PET, and PS mixtures between 0.5 and 850 μg each was tested, referring to about 0.001–0.1% of the plastics in 1 g of soil. For PET, we found that at concentrations of 0.01–0.1%, vinyl benzoate is recommendable for quantification, while ethyl benzoate is better for testing a lower limit of quantification (0.001–0.01%), as higher sensitivity over vinyl benzoate was achieved.

Responses were linear for PE and PET at the highest tested concentration (850 μg), with coefficients of determination (*R*^2^) of 0.94 and 0.92, respectively. However, for PS, the signal response was not linear at concentrations over 120 μg (Figure S5, SI). Therefore, a double log calibration^36^ was made for PS to correlate a linear relationship of concentration ranges over multiple orders of magnitude (see PS2, Table S2, Figure S3, SI), resulting in a good fit (*R*^2^ = 0.97). From personal observation, while calibrating PS in matrices such as sand and soil, responses become much linear at higher concentrations as signals overall are suppressed within a matrix, as was the case with Dierkes et al. who demonstrated a linear range of PS between 0.005 and 1 mg g^–1^ in sand.^23^

The quantification of plastics at low concentrations was until now challenged by the poor solubility of some plastic polymers (PE, PET), limitations in weighing small amounts (<10 μg), and the pyrolysis process. According to Lauschke et al., tests with labeling of environmental samples by adding deuterated styrene showed high variability of recovery due to partial loss of the isotope label during pyrolysis.^25^ We solved the problem of weighing by using precisely cut, volume-defined particles out of our plastic standard materials and used these for lower limit calibration.^26^ The lower limit of calibration was linear for PE (*R*^2^ = 0.98), PET (*R*^2^ = 0.93), and PS (*R*^2^ = 0.92) and stayed in accordance with Dierkes et al. who reported an *R*^2^ of 0.98 for PE and 0.99 for PS using a calibration range from 0.005 to 10 mg in a Py-GC-MS system.^23^

### Method Development—Comparison of Plastic Specific Signals to Organic Materials

In a soil matrix, separation of plastic pyrolysis products from interferences with other OM was the most critical. We could exclude interferences from OM for PS and PET in our method; however, for PE pyrolysis products, tetradecadiene and pentadecadiene, OM interference had to be considered.

The PE pyrolysis compounds 1,13-tetradecadiene and 1,14-pentadecadiene were previously suggested to be suitable for quantifying PE in soils.^18,23^ However, previous potential interference of petroleum with PE detection was already discussed.^23^ In our study, we checked fresh biomass (wood, yeast) and recalcitrant organic materials (humic acid and leonardite) and for the latter found significant contributions of alkadienes (1,13-tetradecadiene was on average 0.8% and 1,14-pentadecadiene was 2% of our humic reference materials, Table S3, SI). This can be explained by diagenetic alteration of organic compounds, i.e., humic acid formation in soil and coal contributions from geological materials may accumulate kinetically stable organic carbon forms resisting oxidation^37^ and yielding alkenes and alkadienes similar to PE from pyrolysis. Hence, as in soil, pedogenic, geogenic, and anthropogenic sources of recalcitrant OM are present, and a quantification method for PE must consider this.

When studies aim at an overall quantification of a variety of plastic types, we suggest correcting the quantification of PE based on pyrolysis and alkadiene detection by the average amount of tetradecadiene detected in humic acids and leonardite, as pentadecadiene had double the contribution from humic materials. We here estimated that all soil OM consist of humic substances; however, analyses of recalcitrant carbon forms in the respective samples should allow a more realistic estimate. We thus provide here a conservative correction for PE content analyses in soil.

### Plastic Detection in Soil Using TD-GC-MS/MS

We tested plastic detection and quantification for two agricultural soils, a sandy silt and a clayey sand, with comparable pH and organic carbon contents of 1.1 and 1.5%, respectively (see the analytical scheme in Figure 1 and Tables 1, S1, SI). PET was detected in both soils, much higher in sandy silt, while PS was under the LOQ in clayey sand and in low amounts in sandy silt (Table 2). For PE, we estimated a total amount of 0.4 and 0.6 mg g^–1^ in sandy and silty soil, respectively (Table 2), when using pentadecadiene for quantification as suggested by, e.g., Dierkes et al.^23^ The advantage of tetradecadiene is that the humic material contribution was lower compared to that of pentadecadiene. However, when organic carbon contents in a soil matrix were above 15 mg g^–1^, there was too much interference to separate tetradecadiene from other compounds and cleanup steps should be considered. As for pentadecadiene, the overestimation factor was much higher; the total PE contents detected in the clayey sand were lower than the potential overestimation by OM; and a PE_corrected_ could not be estimated (Table 2). In the silty soil, the variation of the pentadecadiene was high due to weak peak separation, and a relation to tetradecadiene was not obvious, and although correction worked, both indicated that organics contributed substantially (Table 2 and example calculation in the SI). Finally, we showed that OM could contribute a maximum of 72% to the PE quantification compound (Table 2). Hence, we expect that previous studies using pyrolysis or TD-GC-MS for river sediments^33^ and suspended particulate matter^18,22,35^ overestimated PE contents. While PET and PS were well-quantifiable with our method, for PE, we still recommend either a correction for contribution by OM contents or a partial removal by solvent extraction^17,23^ or density fractionation.^21,38^

**Table 2:** Quantification of Microplastics via Solid Standard Addition to Two Soil Types^a^

			PE_corrected_^a^ quantified via		
soil^b^	OC [g kg^–1^]	PET [mg kg^–1^]	1,13-tetradecadiene [mg kg^–1^]	1,14-pentadecadiene [mg kg^–1^]	PS [mg kg^–1^]
clayey sand	10.71	166.4 ± 17.0	67.7 ± 35.7 (245.3 ± 35.1)	<potential OM contribution (389.3 ± 142.3)	<LOQ (0.31)
sandy silt	15.44	2670.1 ± 415.9^c^	OC too high	273.3 ± 159.4^c^ (605.3 ± 159.0)^c^	20.8 ± 3.9

a For PE, we provide a corrected estimation and the uncorrected, “total PE” amount in brackets.

b 1 g sample aliquot, unless otherwise noted.

c Quantified with a 0.5 g sample aliquot.

This TD-GC-MS/MS method can be adapted further by including other plastics and marker compounds, such as tire and road wear, that has only recently been approached for lake sediments and road dust.^29,39^ We did tests for PP, PA66, PMMA, PLA, and PBAT and see high potential to extend this method to a rather complete variety of plastics (see Table S4, SI).

To summarize, a novel offline large-volume pyrolysis adsorption–thermal desorption–GC-MS/MS method was developed to simultaneously evaluate PS, PE, and PET in larger (>1 g) dried soil samples down to a concentration of 1 mg kg^–1^, allowing representative analyses of plastic concentrations in homogeneous environmental samples and for larger areas such as agricultural topsoil. Rectangular, volume-defined standard particles (125 × 125 × 20 μm^3^) were developed for calibration and lowered the LOQ for PS, PE, and PET than was possible with previous particles (200–400 μm) due to their lower individual weight. For two soil types, sand and silt, a standard addition method was able to quantify PS, PET, and PE_corrected_ in a complete soil matrix, highlighting that plastic quantification in agricultural soil is feasible without any sample cleanup except for interferences of PE with OM.

Of the organics tested, the recalcitrant materials showed relevant contributions to PE quantification, which was highest for 1,14-pentadecadiene compared to 1,13-tetradecadiene. Hence, we proposed a correction for PE and can show that an overestimation of PE contents of up to 70% might appear in environmental studies. For PE detection in samples containing >1.5% organic carbon, correction was not applicable and cleanup is required. As the average agricultural topsoil in temperate regions has 0.9% organic carbon (e.g., Steinmann et al.),^40^ we suggest to limit sample cleanup for PE to soils rich in OM and depending on the relevance of PE for the specific research question. If PE quantification would be in the focus of research and cleanup is required, i.e., the removal of OM by density separation, digestion, and organic solvent extraction, we recommend follow-up studies to check for underestimation of PE and other plastics due to potential loss.

We here established a method that builds the base for quantification of various plastic types in complex environmental samples such as biological tissues, sediments, water, and soils. For bringing the method into widespread application in soil science, soils with different properties should be tested, e.g., differing amounts and quality of OM and reactive minerals, to further ensure the method robustness against matrix effects and finally study plastic concentration, composition, and spatial heterogeneity in soils from many geographical and agricultural contexts.

## Supporting Information Available

Soil information, calibration slopes and equations, organic contributions, additional TD-GC-MS/MS analysis of plastic polymers (PLA, PA, PMMA, PP, PBAT), and example calculations (PDF)
