# Supplementary material for: Plastic Quantification and Polyethylene Overestimation in Agricultural Soil Using Large-Volume Pyrolysis and TD-GC-MS/MS
Source: Environ Sci Technol. 2024 Jul 8;58(29):13047–55. doi: 10.1021/acs.est.3c10101 (PMC11270980; doi:10.1021/acs.est.3c10101)
Supplement: Supplementary file 1 — es3c10101_si_001.pdf [file es3c10101_si_001.pdf]

Plastic Quantification and Polyethylene Overestimation in Agricultural Soil Using Large-Volume Pyrolysis and TD-GC-MS/MS

Ryan Bartnick*[1], Andrei Rodionov[1], Simon David Jakob Oster[2], Martin G. J. Löder[2], Eva Lehndorff[1]

[1]Soil Ecology, University of Bayreuth, Dr.-Hans-Frisch-Str. 1-3, 95448 Bayreuth, Germany

[2]Animal Ecology I, University of Bayreuth, Universitätsstraße 30, 95447 Bayreuth, Germany

*Corresponding author: Email: ryan.bartnick@uni-bayreuth.de

This supporting information file includes:

Table S1-S4 p. S2-S5

Figure S1-S5 p. S6-S10

Calculation examples for PE quantification accounting for OM contribution p. S11

Supporting Information

The SI contains soil information, calibration slopes and equations, organic contributions, additional TD-GC-MS/MS analysis of plastic polymers (PLA, PA, PMMA, PP, PBAT), and example calculations. The data that support the findings of this study are public and openly available within the CRC 1357 Microplastics community on Zenodo at doi.org/10.5281/zenodo.6563563

Table S1. Basic measured parameters of soil investigated (triplicates)

	sand	silt
classification (WRB)[a]	sandy loam (SL)	silt loam (SiL)
sand (%)[b]	78.5	22.5
silt (%)[b]	9.7	63.7
clay (%)[b]	11.8	13.8
pH value	6.7	6.5
total C [g kg^{-1}][c]	10.92	15.54
$C_{organic}$ [g kg^{-1}][d]	10.71	15.44
$C_{inorganic}$ [g kg^{-1}]	0.21	0.10
total N [g kg^{-1}][c]	0.95	1.57

[a]*World reference base for soil resources 2014: International soil classification system for naming soils and creating legends for soil maps [3. ed.]. (2014). World soil resources reports: Vol. 106. FAO.*

[b]*Particle size analyzed from PARIO (Meter Group, Munich, Germany) automated soil particle size analysis.*

[c]*C and N measurements analyzed from elemental analyzer Vario Max CN (Elementar, Langenselbold, Germany).*

[d]*Organic C derived from loss on ignition (combustion at 550 °C for 12 h) and total C analysis.*

Table S2. Pure plastic polymers calibrated limit of quantification (LOQ), slope, response (x = polymer concentration in μg), coefficient of determination (R^2), and tested range[a]

polymer label	LOQ [µg]	y = slope*x + (blank offset)	R^2	log(y) = slope*log(x) + log(b)	R^2	tested range [μg]
PET1	–	–	–	2.10 * log(x) – 2.28	0.92	0.50 - 850
PET2[b]	0.50	1569470 * x + (74615)	0.93	–	–	0.50 - 850
PE2[b]	0.96	281851 * x + (1869706)	0.98	0.69 * log(x) + 3.91	0.94	0.96 - 850
PE3	0.96	222838 * x + (997975)	0.96	0.87 * log(x) + 2.80	0.91	0.96 - 850
PS2[b]	0.31	22986388 * x + (24570)	0.92	0.90 * log(x) + 4.59	0.97	0.31 - 850

[a]*Double log(x,y) combining low and high concentration calibrations in a power curve to a linear function to account for multiple levels of magnitude.*

[b]*Used for determining LOQ.*

Table S3. Plastic quantification compound interferences from different organic materials[a]

polymer label	compound (pyrolysis product)	wood	yeast	leonardite (HT)	humic acid (HT)	humic acid (SA)	PE overestimation (average)[b]
PET2	ethyl benzoate [μg mg^{-1}]	< 0.50	< 0.50	< 0.50	< 0.50	< 0.50	-
PE2	1,13-tetra-decadiene [μg mg^{-1}]	< 0.96	< 0.96	12.0 ± 1.4	10.6 ± 0.4	2.1 ± 0.3	8.2 ± 4.7
PE3	1,14-penta-decadiene [μg mg^{-1}]	< 0.96	< 0.96	15.3 ± 2.5	38.2 ± 2.8	10.5 ± 1.0	21.3 ± 13.0
PS2	2,4-diphenyl-1-butene [μg mg^{-1}]	< 0.31	< 0.31	< 0.31	< 0.31	< 0.31	-

[a]*Humic materials were provided by Humintech (HT) and Sigma-Aldrich (SA).*

[b]*"PE overestimation" mean and standard deviation in µg plastic signal per mg organic substance (20 mg, n = 3).*

Table S4. Additional plastic polymer compounds from pyrolysis products of plastics polypropylene (PP), polyamide 66 (PA66), polymethyl methacrylate (PMMA), and biodegradable polymers polylactic acid (PLA) and polybutylene adipate terephthalate (PBAT)[a]

polymer label	compound (pyrolysis product)	t_R (min)	molecular formula	molar mass	SRM (*m/z*)	
					Q1	Q3
PLA	lactide	12.0 – 12.9	$C_6H_8O_4$	144	56	28
PA66	caprolactam	14.6	$C_6H_{11}NO$	113	113	85
PMMA	methyl methacrylate	14.9	$C_5H_8O_2$	100	69	41
PP	2,4,6,8-tetramethyl-10-undecene	15.9 - 16.3	$C_{15}H_{30}$	210	111	69
PBAT	terephthalic acid dibut-3-enyl-ester	27.6	$C_{16}H_{18}O_4$	274	203	149

[a]*Compounds identified by specific retention time (t_R) and selected reaction monitoring (SRM) ions of interest (m/z) at quadrupole 1 (Q1) and quadrupole 3 (Q3) of MS/MS.*

Additional polymers: Using the same deductive method development (SIM to PIS to SRM), we additionally analyzed more plastic polymer types to provide a foundation for MS/MS application for plastics analysis. These results are the first analysis of biodegradable polymers analyzed by MS/MS and, by using our method, can be used as starting point to simultaneously detect many plastic types in soil and environmental matrices which are of interest to researchers. All these plastic pyrolysis products can be separated by retention time in the chromatographic column with minimal overlap to allow simultaneous detection.

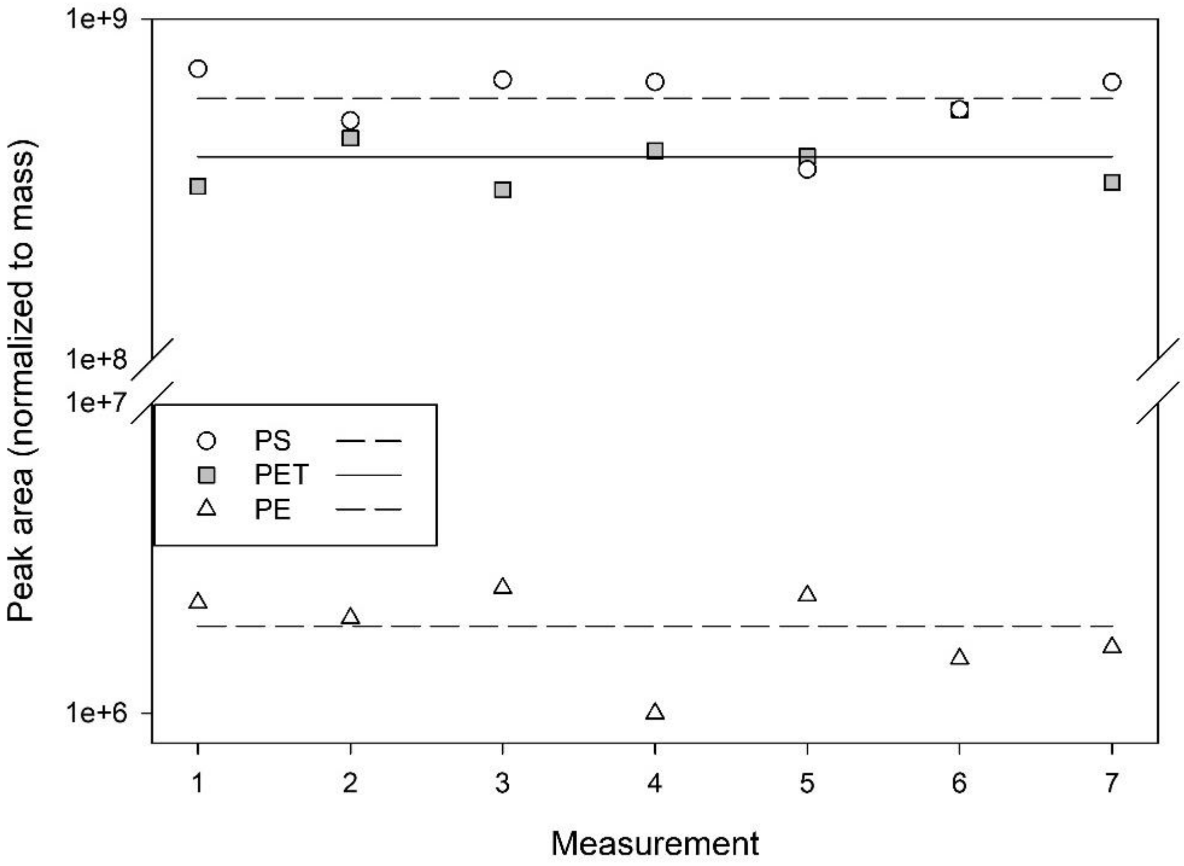

Figure S1. Deviation in area of mass fragment peaks for calibration compounds (7 replicates) of polystyrene (PS2; relative standard deviation, RSD, 19%), polyethylene terephthalate (PET1, RSD 19%), and polyethylene (PE2, RSD 27%), with averages (straight lines). Mix of pure polymers: 200 μg each, normalized to the sample mass.

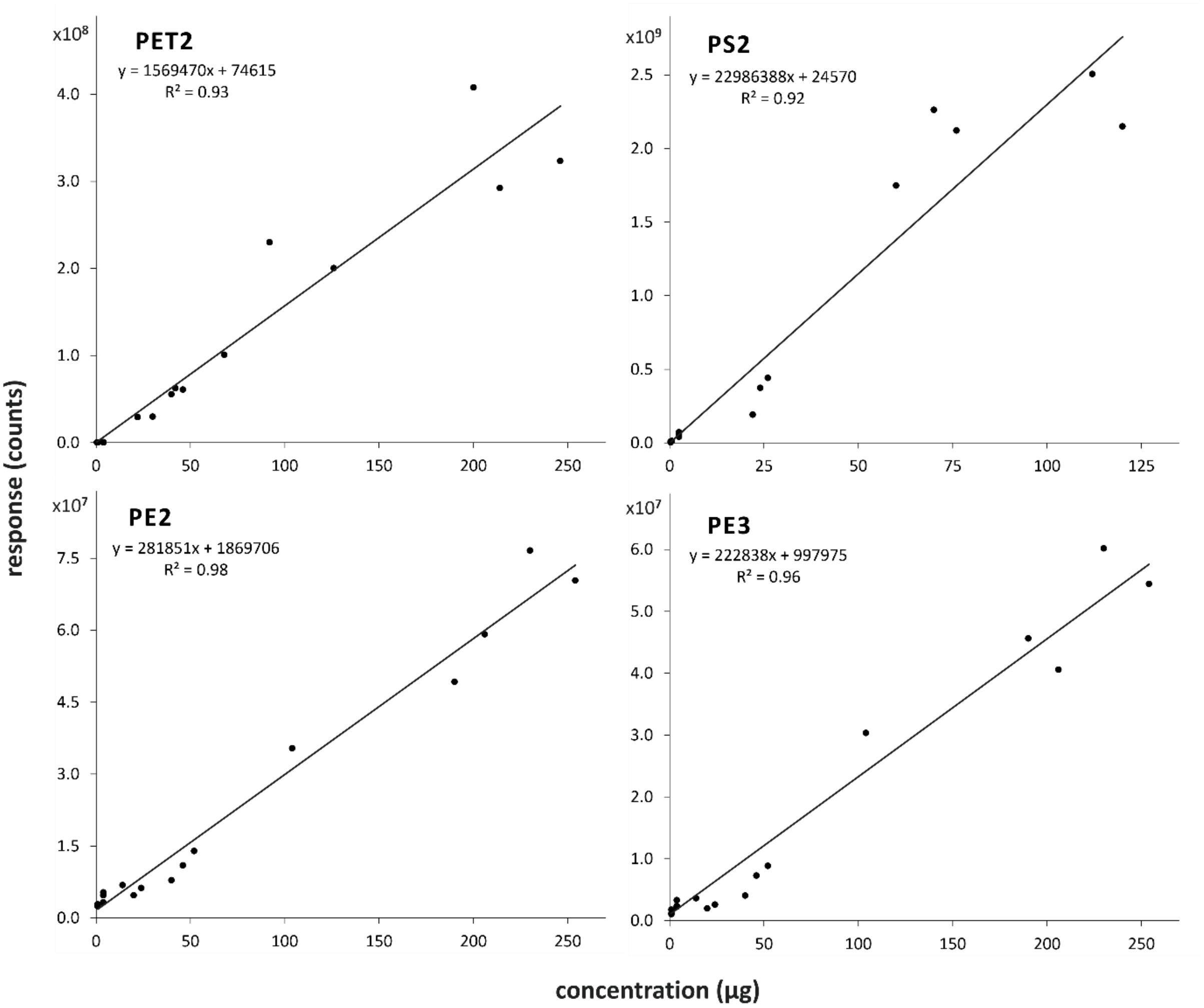

Figure S2. Calibration curves of lower plastic concentrations (*n* = 20, each plastic mixed) for determining limit of quantification; showing concentration vs. response for a mix of polyethylene (PE), polyethylene terephthalate (PET), and polystyrene (PS) with standards in the concentration range approx. 0.5 to 250 μg.

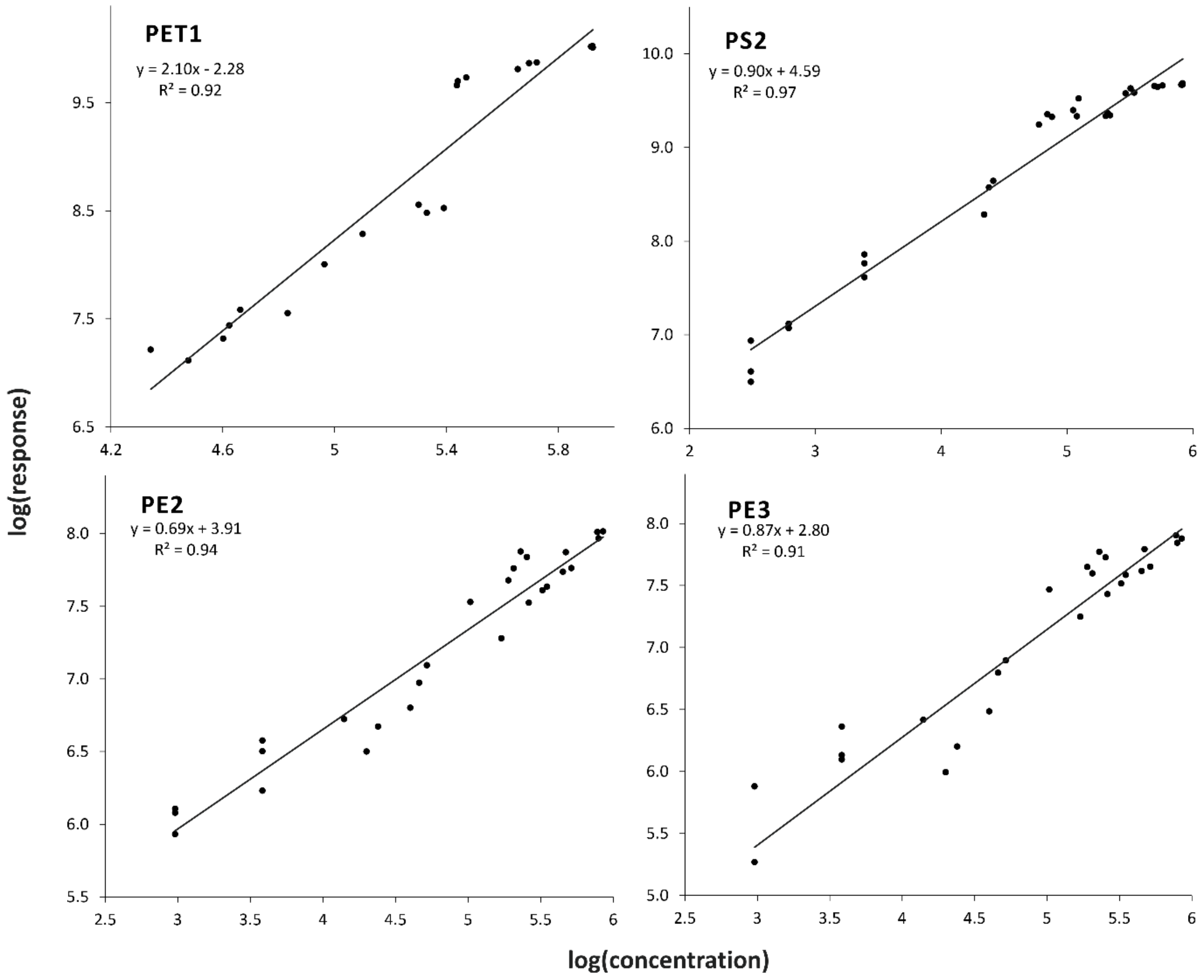

Figure S3. Double log plot of plastic concentrations vs. response over multiple levels of magnitude, approx. 0.5 to 850 μg (n = 30, each plastic mixed), to calibrate a linear function from a power curve (calibration for a large concentration range of plastic, e.g. for samples with yet unknown plastic contents). Note that calibration depends on actual system performance and must be checked regularly.

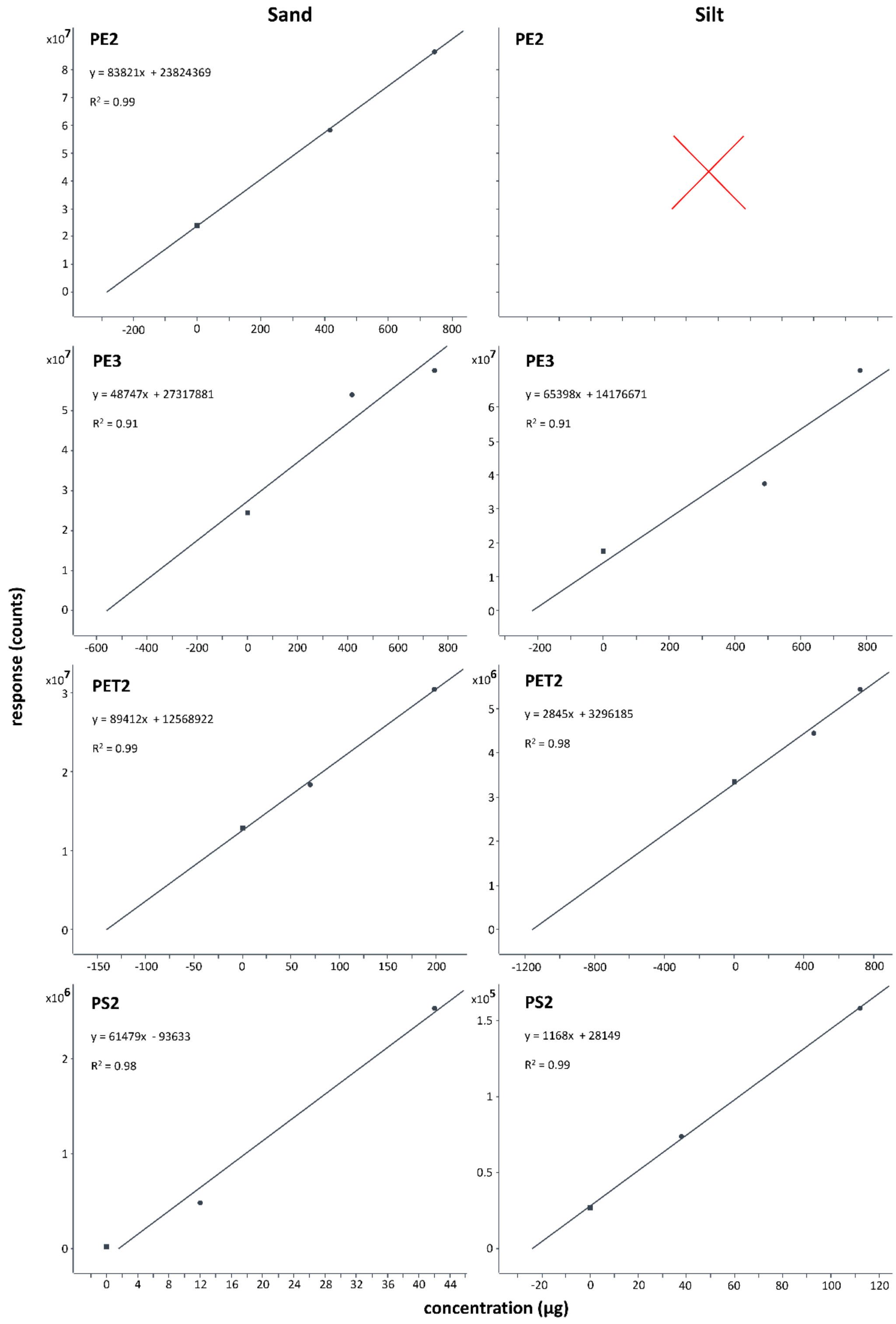

Figure S4. Standard addition three-point calibration for quantification of microplastic in sand and silt agricultural soils. As the magnitude of plastic concentration in soil is unknowable before testing, "spiked" standard additions should first be made in the magnitude of expected quantification, then adjusted until additions are within the sample analyte concentration range for each polymer tested. PE2 in silt soil was not distinguishable from peak separation in sample.

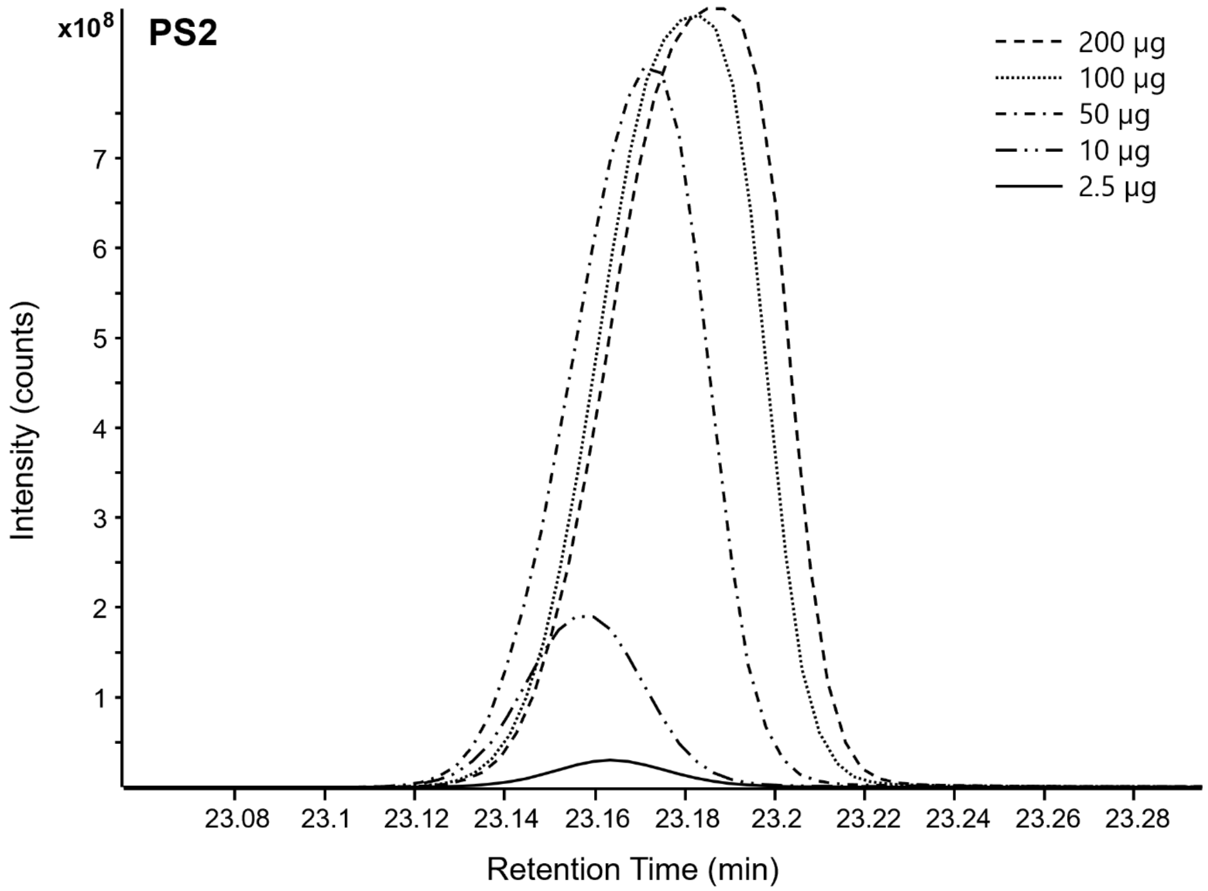

Figure S5. MS/MS chromatogram of PS dimer peak broadening at higher concentrations.

Calculation examples for PE quantification accounting for OM contribution:

(soil PE concentrations in manuscript were calculated from analysis of 5 replicates)

From averaged OM signals (Table S3, µg mg^{-1} converted to *w/w*):

$$PE_{overestimation\ factor} = \frac{(\text{alkadiene}_{\text{OM}}\ [\text{AU}] \times \text{OM}\ [\text{SI}])}{(\text{alkadiene}_{\text{PE}}\ [\text{AU}] \times \text{PE}\ [\text{SI}])} \quad \text{[Eq. 1]}$$

1,13-tetradecadiene: $PE_{overestimation\ factor}$ = 0.00824

1,14-pentadecadiene: $PE_{overestimation\ factor}$ = 0.02134

$$\text{PE}_{\text{corrected}}\ [\text{mg g}^{-1}] = \text{PE}_{\text{soil}}\ [\text{mg g}^{-1}] - \left(\text{sample}\ [\text{g}] \times 2\left(\text{OC}_{\text{sample}}\ [\text{mg g}^{-1}]\right) \times PE_{overestimation\ factor}\right) \quad \text{[Eq. 2]}$$

Sandy:

1,13-tetradecadiene:

$PE_{corrected}$ = 0.2453 [mg g^{-1}] – (1.006 [g] * 2(10.71 [mg g^{-1}]) * 0.00824 [factor])

= 0.2453 – (0.1776) = 0.0677 [mg g^{-1}] = **67.7 [µg g^{-1}]**

$PE_{overestimation}$ = 0.1776 [mg g^{-1}] = **177.6 [µg g^{-1}]**

1,14-pentadecadiene:

$PE_{corrected}$ = 0.3893 [mg g^{-1}] – (1.006 [g] * 2(10.71 [mg g^{-1}]) * 0.02134 [factor])

= 0.3893 – (0.4599) = -0.0706 [mg g^{-1}] = **-70.6 [µg g^{-1}] negative, potential contribution by OM higher than signal intensity**

$PE_{overestimation}$ = 0.4599 [mg g^{-1}] = **459.9 [µg g^{-1}]**

Silty:

1,13-tetradecadiene: Too much interference from OM (OC >1.5%), no clear peak separation

1,14-pentadecadiene:

$PE_{corrected}$ = 0.6053 [mg g^{-1}] – (0.504 [g] * 2(15.44 [mg g^{-1}]) * 0.02134 [factor])

= 0.6053 – (0.3321) = 0.2732 [mg g^{-1}] = **273.2 [µg g^{-1}]**

$PE_{overestimation}$ = 0.3321 [mg g^{-1}] = **332.1 [µg g^{-1}]**

Red color: overestimation by organic materials
